# Identification of an ERK Inhibitor as a Therapeutic Drug Against Tau Aggregation in a New Cell-Based Assay

**DOI:** 10.3389/fncel.2019.00386

**Published:** 2019-08-21

**Authors:** Giacomo Siano, Maria Claudia Caiazza, Ivana Ollà, Martina Varisco, Giuseppe Madaro, Valentina Quercioli, Mariantonietta Calvello, Antonino Cattaneo, Cristina Di Primio

**Affiliations:** ^1^Laboratorio di Biologia (BIO@SNS), Scuola Normale Superiore, Pisa, Italy; ^2^Neurotrophins and Neurodegenerative Diseases Laboratory, Rita Levi-Montalcini European Brain Research Institute, Rome, Italy

**Keywords:** Tau, CST, Tau biosensor, FRET, ERK, aggregation

## Abstract

Formation of Tau aggregates is a common pathological feature of tauopathies and their accumulation directly correlates with cytotoxicity and neuronal degeneration. Great efforts have been made to understand Tau aggregation and to find therapeutics halting or reversing the process, however, progress has been slowed due to the lack of a suitable method for monitoring Tau aggregation. We developed a cell-based assay allowing to detect and quantify Tau aggregation in living cells. The system is based on the FRET biosensor CST able to monitor the molecular dynamic of Tau aggregation in different cellular conditions. We probed candidate compounds that could block Tau hyperphosphorylation. In particular, to foster the drug discovery process, we tested kinase inhibitors approved for the treatment of other diseases. We identified the ERK inhibitor PD-901 as a promising therapeutic molecule since it reduces and prevents Tau aggregation. This evidence establishes the CST cell-based aggregation assay as a reliable tool for drug discovery and suggests that PD-901 might be a promising compound to be tested for further preclinical studies on AD.

## Introduction

Tauopathies are complex multifactorial diseases (Lee et al., 2001; Wang and Mandelkow, 2016) and, up to now, despite strong efforts, the drug discovery process has been inconclusive. Current approaches for the treatment of these pathologies do not rely on disease modifying drugs but rather on symptomatic treatments with the aim of attenuating and delaying behavioral degeneration (Birks, 2006; Wang and Reddy, 2017).

However, these treatments cannot halt the progress of the pathology and the scientific community is working on therapeutic approaches aimed at preventing the development and progression of neurodegeneration by targeting the toxic amyloidogenic aggregates (Devos et al., 2017; West et al., 2017; Novak et al., 2019).

Tau protein is considered a promising candidate since several molecular mechanisms leading to Tau aggregation are characterized and may be specifically targeted. Among Tau-directed drugs, molecules affecting Tau hyperphosphorylation are the most interesting since abnormal phosphorylation is strongly associated to pathological Tau destabilization from microtubules, structural alteration and aggregates formation (Alonso et al., 1994, 2001; Pei et al., 2003; Pevalova et al., 2006; Medina and Avila, 2015; Wang and Mandelkow, 2016).

Many different kinase inhibitors have been approved for the treatment of other diseases such as cancer. Among these, PD-0325901 (here reported as PD-901) and D-JNKI-1 may be repurposed for the treatment of tauopathies. PD-901 is a potent inhibitor of ERK pathway since it inhibits MEK1 and MEK2 preventing the activation of ERK (Barrett et al., 2008; Henderson et al., 2011; Yap et al., 2011). It is currently in clinical trial for the treatment of lung cancer and solid tumors (ClinicalTrials.gov Identifier: NCT02022982; NCT02039336; NCT03905148). D-JNKI-1 is a cell-penetrating peptide that inhibits JNK pathway (Hirt et al., 2004; Milano et al., 2007) and it is currently in clinical trial for the treatment of acute hearing loss (ClinicalTrials.gov Identifier: NCT02809118; NCT02561091).

These molecules could be of great interest also to treat tauopathies since both ERK and JNK pathways have been demonstrated to target Tau specific residues resulting in hyperphosphorylation and aggregation enhancement (Perry et al., 1999; Zhu et al., 2001; Hanger et al., 2009).

Nowadays a crucial point for the drug discovery against tauopathies is the development of cell-based molecular tools for preclinical screening and testing of potential therapeutic drugs. Several biosensors to study Tau aggregation have been proposed (Kfoury et al., 2012; Tak et al., 2013).

Recently, we developed the Conformational Sensitive Tau (CST) sensor, the first intramolecular FRET-based biosensor allowing to discriminate Tau full length conformations depending on its association to microtubules, its release in the cytosol and its aggregation (Di Primio et al., 2017).

Here we report a CST-based cellular screening to test potential therapeutic molecules against Tau aggregation. The screening exploits the differentiated SH-SY5Y cell line, stably expressing CST reporter, where Tau aggregation is induced by synthetic Tau seeds. We tested two kinase inhibitors, PD-901 and D-JKNI-1 and remarkably we found that PD-901, more than D-JNKI-1, is able to interfere with Tau aggregation. The anti-aggregation activity is retained also in primary hippocampal neurons supporting the preclinical application of the CST cell-based aggregation assay but also the clinical employment of PD-901, not only for the treatment of cancers, but also for tauopathies.

## Results

### CST Cell-Based Aggregation Assay

To set up the CST cell-based aggregation assay, we first validated the biosensor as a sensitive tool in detecting Tau aggregates displaying proteopathic features. Briefly, the CST is the fusion of the Tau protein with the CFP at the C-terminal and the YFP at the N-terminal. In normal conditions, the binding to MTs induces the folding of the CST into a loop-like conformation that brings the fluorophores close together generating the FRET signal; on the contrary, upon aggregation it detaches from MTs and forms FRET-positive inclusions in the cytoplasm without affecting the MT network (Di Primio et al., 2017). Hela cells expressing CST^*P*301*S*^ were exposed to recombinant heparin-assembled Tau seeds to induce aggregation (as described in M&M). The P301S mutant has been preferred to Tau^*WT*^ to enhance its contribution to intracellular aggregates (McEwan et al., 2017). The quantitative sensitized emission FRET microscopy was employed to detect intracellular aggregates 72 h after induction. In control cells we detected the expected FRET signal displayed by CST bound to MTs, as previously reported (Di Primio et al., 2017).

**FIGURE 1:**
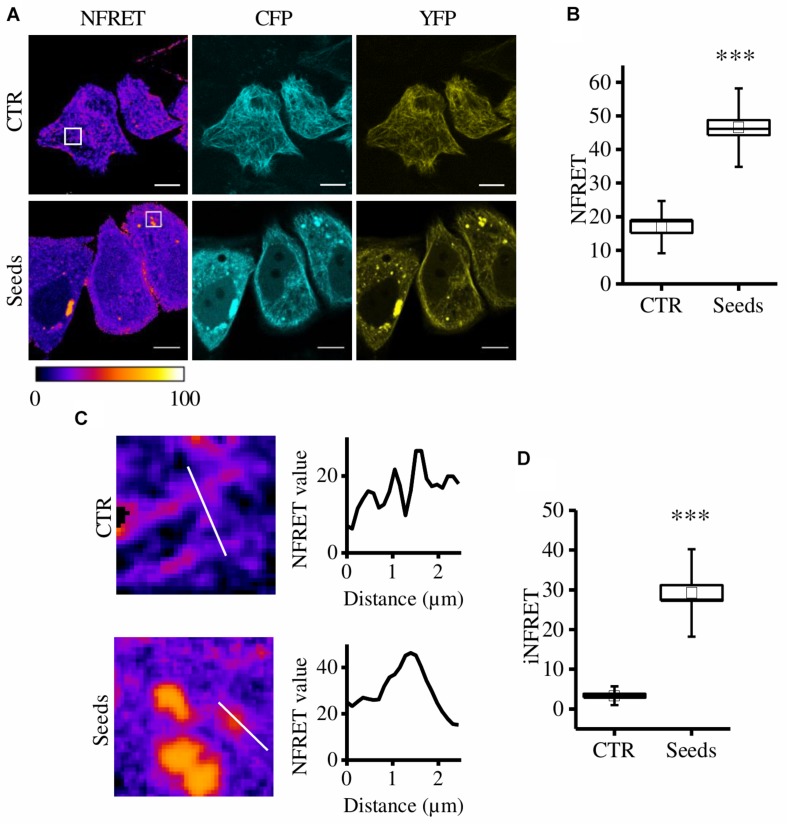
CST aggregates are detected and quantified in live cells. **(A)** FRET measured by sensitized emission in CST-reporter HeLa cells. Donor (cyan), acceptor (yellow) and Normalized FRET (NFRET) images (false color). White scale bar = 10 μm. **(B)** Box plot of NFRET values calculated on MTs network (*N* = 9) and on aggregates (*N* = 12). Box spans the standard error of the mean while whiskers indicates the standard deviation (^∗∗∗^*p* < 0.001 *t*-test). **(C)** Magnification of cells in A and corresponding NFRET profile along the white line crossing MTs (upper panel) or aggregates (lower panel). **(D)** Box plot of iNFRET values calculated on MTs network (*N* = 9) and on aggregates (*N* = 19). Box spans the standard error of the mean while whiskers indicates the standard deviation (^∗∗∗^*p* < 0.001 *t*-test).

On the contrary, in cells exposed to Tau seeds, the FRET signal was not associated to MTs but discrete FRET-positive spots were detectable in the cytosol (Figure 1A).

By comparing cells exposed to seeds and control cells we found that NFRET values on aggregates were significantly higher (46.50 ± 2.25SE) than those on microtubules (16.89 ± 1.73SE) indicating that Tau molecules interaction in aggregates is tighter (Figure 1B). Moreover, this difference could be also due to the higher amount of molecules contributing to the FRET signal.

To take into account at the same time the close interaction among Tau molecules and the differences in size of aggregates, a line crossing either MTs or aggregates was selected, the corresponding NFRET profile was obtained and the integral below was designed to be the integrated NFRET signal (iNFRET) (Figure 1C). iNFRET values on aggregates were significantly higher (30.84 ± 2.49) than those on microtubules (3.35 ± 0.52) highlighting the larger size of aggregates with respect to MTs (Figure 1D).

**FIGURE 2:**
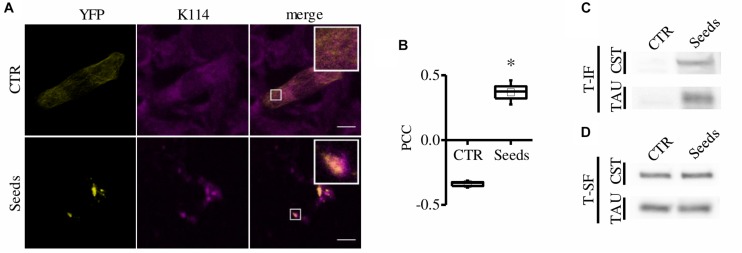
CST aggregates are amyloidogenic proteopathic aggregates. **(A)** CST-reporter HeLa cells were stained with K114. YFP (yellow), K114 (magenta), white scale bar = 10 μm. **(B)** Colocalization between CST and K114 signals has been quantified by Pearson Correlation Coefficient (PCC). Box spans the standard error of the mean while whiskers indicates the standard deviation (^∗^*p* < 0.01 *t*-test). **(C–D)** Detergent fractionation of CST-reporter and unlabeled Tau-reporter cells. Western blot of the Triton-X100 insoluble fraction (T-IF) and of the Triton-X100 soluble fraction (T-SF) developed with the Tau5 antibody.

To further verify that CST-positive spots correspond to amyloidogenic proteopathic aggregates, we performed the K114 staining for β-sheet fibrils (Crystal et al., 2003). Remarkably, CST signal significantly colocalizes with K114 (Costes et al., 2004) (Figures 2A,B) demonstrating that FRET-positive aggregates share structural characteristics with pathological Tau fibrils.

To investigate the putative interference of the CST fluorophores on aggregate FRET signal, we performed an aggregation assay exploiting cells expressing unlabeled Tau^*P*301*S*^. Cells were added with recombinant heparin-assembled Tau fibrils and aggregates were visualized by immunofluorescence. We found that aggregates in CST- or in unlabeled Tau- reporter cells displayed similar morphology, demonstrating that the fluorophores do not interfere with the process of aggregation (Supplementary Figures S1A,B).

Finally, we performed detergent fractionation to investigate possible biochemical differences in these aggregates. We found that both CST and Tau increased in Triton X-100 insoluble fraction upon aggregation induction (Figure 2C).

Altogether these results indicate that, upon aggregation induction, CST forms FRET positive inclusions that display morphological and biochemical features allowing them to be defined as Tau aggregates.

**FIGURE 3:**
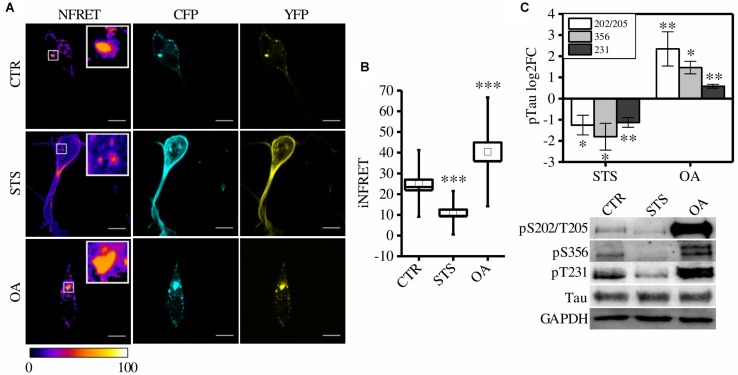
CST aggregates are altered by phosphorylation in SH-SY5Y cells. **(A)** FRET measured by sensitized emission in CST reporter cells treated with seeds (CTR) or treated with seeds and STS or OA. CFP (cyan), YFP (yellow) and Normalized FRET (NFRET) images (false color). White scale bar = 10 μm. **(B)** Box plot of iNFRET values calculated in CTR cells (*N* = 37), STS treated cells (*N* = 21) and OA treated cells (*N* = 15). Box spans the standard error of the mean while whiskers indicates the standard deviation (^∗∗∗^*p* < 0.001 ANOVA one-way test). **(C)** WB and corresponding quantification of phosphorylation at indicated epitopes in CST reporter cells treated with STS or OA (*N* = 4).

To exploit the system as a quantitative tool to measure Tau aggregation in different cellular conditions a CST^*P*301*S*^-reporter line has been generated in a neuron-like cellular model, SH-SY5Y cells. Reporter cells were differentiated and exposed to synthetic Tau^*P*301*S*^ seeds to induce aggregation. FRET positive aggregates were easily detectable in seeds-treated cells (hereinafter referred as “control cells, CTR”) as reported in Figure 3A with a value of iNFRET that is comparable to that obtained in HeLa cells (25.19 ± 1.77SE). The SH-SY5Y cellular set-up has been exploited for the following functional experiments.

It is well known that Tau hyperphosphorylation results in aggregation that can be further exacerbated by boosting phosphorylation (Pei et al., 2003; Despres et al., 2017).

To test whether CST^*P*301*S*^-reporter cells could detect these modulations, cells were treated with drugs inhibiting or promoting phosphorylation: Staurosporine (STS) and Okadaic acid (OA), respectively. STS is a potent, non-specific protein kinases inhibitor which prevents Tau phosphorylation; on the contrary, OA is an inhibitor of protein phosphatase PP2A which induces Tau hyperphosphorylation (Rüegg and Burgess, 1989; Pei et al., 2003; Karaman et al., 2008; Gani and Engh, 2010).

Seventy-two hours after aggregation induction, cells have been treated with STS or OA. We found that few small aggregates appeared in STS treated cells but the relative iNFRET values were significantly reduced compared to untreated cells, indicating smaller and less stable aggregates (Figures 3A,B).

On the contrary, by boosting phosphorylation with OA, bigger FRET positive aggregates formed. The increased iNFRET signal confirmed that hyperphosphorylation enhances the accumulation of Tau proteins into aggregates (Figure 3).

Accordingly, the K114 signal was significantly lower in STS-treated cells and higher in OA-treated cells compared to control cells (Supplementary Figure S2).

To further confirm the effect of drugs on Tau phosphorylation we checked by WB the Tau sensitive epitopes T231, S356 and S202/T205 and we found a decreased signal in STS treated cells with respect to OA-treated cells (Figure 3).

Taken together, these findings confirm that the modulation of phosphorylation is quantitatively detected by the FRET signal, with a broad dynamic range, and demonstrate that this system can be a valuable and reliable tool to quantify aggregation in live cells.

### PD-901 Reduces Tau Aggregation

We exploited the CST cell-based screening assay to test candidate therapeutic molecules supposed to reduce Tau aggregation. We tested two FDA-approved kinase inhibitors: PD-901, an ERK inhibitor, and D-JNKI-1, a JNK1 inhibitor.

**FIGURE 4:**
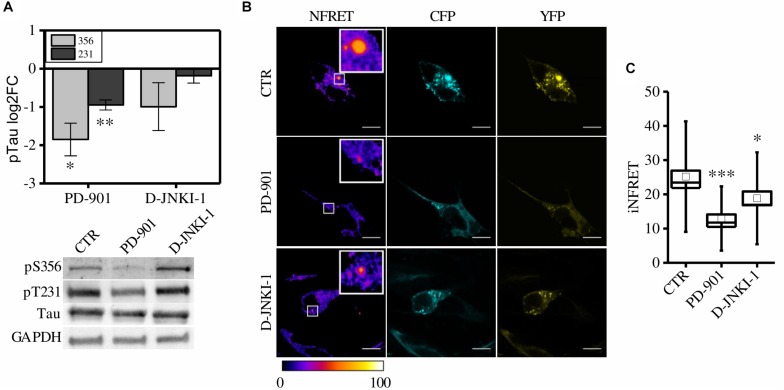
CST aggregates are reduced by PD-901 and D-JNKI-1 treatment in SH-SY5Y cells. **(A)** WB and corresponding quantification of phosphorylated epitopes S202/T205, S231, S356 in CST reporter cells treated with PD-901 or D-JNKI-1 (*N* = 4). **(B)** FRET measured by sensitized emission in reporter cells treated with PD-901 or D-JNKI-1. CFP (cyan), YFP (yellow) and Normalized FRET (NFRET) images (false color). White scale bar = 10 μm. **(C)** Box plot of iNFRET values calculated in CST reporter cells treated with seeds (CTR) (*N* = 37), seeds and PD-901 treated cells (*N* = 27), seeds and D-JNKI-1 treated cells (*N* = 20). Box spans the standard error of the mean while whiskers indicates the standard deviation (^∗∗∗^*p* < 0.001 ANOVA one-way test).

First, to verify the ability of these compound to reduce Tau phosphorylation, control cells pre-exposed to synthetic seeds were treated with PD-901 or D-JNKI-1. The drugs have been used at a concentration that does not induce cytotoxicity (Supplementary Figure S3A). Western blot experiments showed that PD-901 but not D-JNKI-1 decreased Tau phosphorylation at Thr231 and Ser356 residues, as expected (Figure 4A). On the contrary the epitope S202/T205 was unaltered (data not shown).

To investigate the potential *in vivo* anti Tau aggregation effect of the drugs, FRET analysis was employed to quantify aggregation in treated cells (Figure 4B).

We observed, in both PD-901 and D-JNKI-1 treated cells, the formation of small FRET positive aggregates (Figure 4B), however, by measuring the relative iNFRET values, we found that the size and cohesion of aggregates in drug treated cells were significantly reduced compared to control cells. Moreover, the effect of PD-901 in destabilizing Tau aggregates was stronger than D-JNKI-1 (Figure 4C). Indeed, PD-901 reduced the iNFRET to 12.94 ± 1.21 while D-JNKI-1 induced a reduction to 18.82 ± 2.00. Potentially, by blocking aggregation, the drugs might induce the accumulation of Tau soluble species considered even more toxic then big aggregates for the cell. However, the cell toxicity assay (Supplementary Figure S3B) performed in these conditions demonstrated that the viability of treated and untreated cells is comparable. The different effect of drugs on aggregates was confirmed by the K114 staining, reported in Supplementary Figures S3C,D, showing that the amyloid component was significantly lower in PD-901-treated cells compared to control cells. On the contrary, no significant difference can be detected between D-JNKI-1-treated cells and control cells (Supplementary Figure S3D).

**FIGURE 5:**
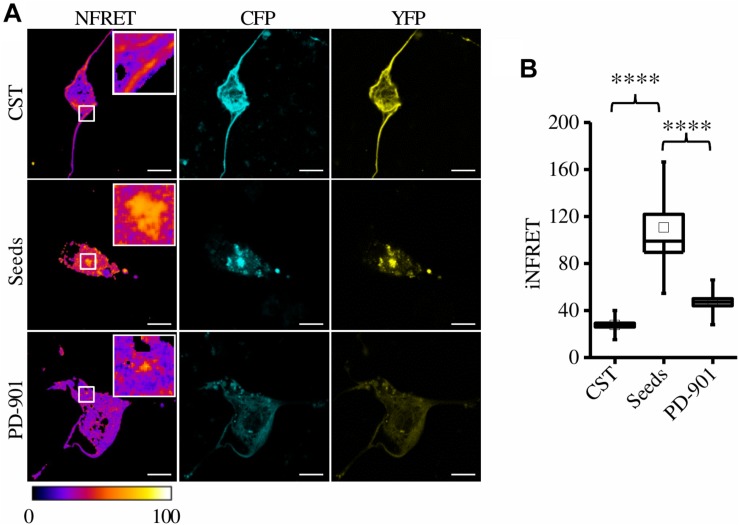
CST aggregates are reduced by PD-901 in mouse primary hippocampal neurons. **(A)** FRET measured by sensitized emission in primary neurons treated with PD-901. CFP (cyan), YFP (yellow) and Normalized FRET (NFRET) images (false color). White scale bar = 10 μm. **(B)** Box plot of iNFRET values calculated in cells expressing CST (*N* = 58), cells treated with seeds (*N* = 24), cells treated with seeds and PD-901 (*N* = 39). Box spans the standard error of the mean while whiskers indicates the standard deviation (^****^p < 0.0001; seed *p* < 0.0000001; PD-901 *p* < 0.00001 ANOVA one-way test).

To further test the anti-aggregation efficacy of PD-901, we treated mouse hippocampal primary neurons expressing the CST^*P*301*S*^ after the exposure to seeds and we performed the FRET experiment. These cells displayed the expected iFRET signals in control and seed-treated conditions (CST: 27.62 ± 1.61; Seeds: 110.54 ± 11.40) (Figure 5). Remarkably, after the treatment with PD-901 the iFRET signal is strongly decreased (PD-901: 47.03004 ± 3.04793) indicating that cohesion and size of aggregates are significantly reduced.

Altogether, these results point out that the cell-based aggregation assay developed is a powerful tool to detect and quantify Tau aggregation. Moreover, we demonstrated that PD-901 could be a potential therapeutic molecule blocking or even reverting Tau aggregation. The assay system might be easily scalable to set up a high content screening assay.

## Discussion

We developed a cell-based assay allowing to detect and quantify Tau aggregation in living cells. The development of a reliable assay for Tau aggregation is necessary not only to identify new therapeutic biomarkers but also to screen potential drug candidates.

In the last 10 years several Tau aggregation systems have been developed to investigate the molecular mechanism in cells. Pouplana et al. (2014) obtained Tau aggregates in a bacterial system, however, this model does not allow post-translational modifications required for Tau aggregation in cells. Several other groups proposed mammalian reporter systems allowing Tau aggregation upon induction with congo red or exogenous Tau fibrils (Bandyopadhyay et al., 2007; Guo and Lee, 2011; Lim et al., 2014). Altogether these studies proved that overexpressed Tau could be aggregated in cells allowing to study the aggregation-induced cellular toxicity. Nevertheless, these approaches required cellular fixation and staining with ThS or antibodies to confirm Tau aggregation. The exploitation of fluorescent proteins allowed to monitor Tau aggregation in living cells. Indeed, by fusing fluorescent proteins to caspase-cleaved Tau or to Tau fragments it is possible to study Tau aggregation and *trans*-cellular propagation of aggregates by FRET microscopy (Rizzo et al., 2004; Chun and Johnson, 2007; Kfoury et al., 2012). Exploiting the bimolecular fluorescence complementation (BiFC) technique, Tau-BiFC sensors have been developed to follow aggregation in real time (Chun et al., 2007, 2011; Tak et al., 2013), however BiFC produces a signal after a delay required for the chemical reactions that generate the fluorophore and this intrinsic property is not appropriate for massive and rapid drug screening.

We previously developed the CST, a FRET-based conformation sensor for full length Tau, forming FRET positive inclusions upon proteopathic Tau seeding (Di Primio et al., 2017). Importantly, this is the first fluorescent sensor exploiting the full length Tau, allowing to study the aggregation and monitoring the increasing or decreasing cohesion of Tau molecules. Since FRET enables instantaneous monitoring of protein interactions, we exploited the CST for a cell-based assay to develop a screening platform for therapeutic compounds.

First we validated the FRET signal of aggregates as related to amyloid inclusions, indeed, they are stained by the K114 dye and are detected in the insoluble fraction after detergent fractionation. Then, since it is generally believed that Tau aggregation is initiated or accelerated by hyperphosphorylation, we checked the modulation of the FRET signal upon inhibition or induction of phosphorylation. We found that the FRET signal of aggregates was quite different after these treatments, the phosphorylation inhibition by STS impaired the aggregation and CST inclusions were very few, small and destabilized. On the contrary, increasing phosphorylation by OA resulted in the appearance of very large and stable aggregates, as expected. This evidence established the CST as a reliable tool for aggregation study in living cells since it closely resembles Tau aggregation dynamic.

In order to propose this system for drug discovery we focused on compounds that could block Tau hyperphosphorylation. Moreover, to foster the identification of active drugs against aggregation we focused on kinase inhibitors already approved for the treatment of cancer or other diseases. These drugs might be used off-label for the treatment of tauopathies with the clear advantage of knowing the working concentrations and the lack of side effects. Among these, the PD-901 and D-JNKI-1 proved to be able to decrease Tau phosphorylation and to reduce Tau aggregation. Moreover, PD-901 decreased phosphorylation at T231 and S356 that are considered sensitive epitopes early modified for the subsequent aggregation (Johnson, 2004).

The analysis of aggregates after treatment with these compounds revealed that both are able to destabilize Tau aggregates, but PD-901 is more efficient than D-JNKI-1 as confirmed also by the K114 staining of β-sheet structures.

Remarkably, as indicated by the Western blot analyses, PD-901 might have a stronger effect by preventing Tau phosphorylation. PD-901 is a pharmacological inhibitor of ERK1/2 acting upstream on MEK1 and MEK2. Interestingly, the ERK pathway seems to target Tau in the very early steps of aggregation, since indeed it phosphorylates key epitopes for subsequent pathological phosphorylations. It is conceivable that in our cell-based assay, the inhibition of ERK activation hampers the domino phosphorylation events, thus preventing the aggregation of Tau soluble molecules. Consistently, the administration of ERK2 inhibitor was able to rescue motor deficits and was able to reduce the levels of abnormally phosphorylated Tau in mouse model of tauopathy (Le Corre et al., 2006; Hanger et al., 2009). On the contrary, the JNK pathway mediates the phosphorylation of several Tau residues that are considered to be involved in the late steps of aggregation and in particular in the stabilization of aggregates (Kins et al., 2003; Johnson, 2004; Hanger et al., 2009; Noël et al., 2015). The weaker effect of D-JNK-1 on aggregation could be explained also by the fact that treated cells showed a level of phosphorylation comparable to untreated cells for the S356 and T231 epitopes. Moreover, this compound could be less easily available to the cell than PD-901.

These findings suggest that PD-901 might be a promising compound to be tested for further preclinical studies on AD and this assumption is strongly corroborated by the encouraging results obtained in primary hippocampal neurons. One major concern about this molecule could be its potential toxicity. In our assay the drug does not alter the viability of cells treated before or after Tau aggregation indicating that it might be active also against small Tau inclusions that are considered even more toxic then big aggregates. However, its toxicity in AD patients need to be assessed.

The use of the CST-based platform will greatly foster and accelerate the drug discovery process and the repurposing of FDA-approved molecules for other diseases might open new perspective in the treatment of tauopathies.

## Materials and Methods

### Cell Culture and Transfection

HeLa cells were routinely cultured in Dulbecco’s Modified Eagle’s Medium (DMEM) low glucose (Euroclone), supplemented with 10% Fetal Bovine Serum (FBS), 100 U/ml Penicillin and 100 μg/ml Streptomycin. SH-SY5Y cells were routinely cultured in Dulbecco’s Modified Eagle Medium: Nutrient Mixture F-12 (DMEM/F-12) (Gibco), supplemented with 10% FBS, 100 U/ml Penicillin and 100 μg/ml Streptomycin. The day before the experiment, cells were seeded at 10^5^ cells per well in six-well plates or in glass bottom dishes (WillCo-dish) or at 10^4^ cells per well in 4- and 8-well formats Chamber Slides (Lab-Tek). DNA transfection in HeLa cells was carried out using Effectene transfection reagent (QIAGEN) according to manufacturer’s instructions. DNA transfection in SH-SY5Y cells was carried out using Lipofectamine 2000 transfection reagent (Thermo Fisher Scientific) according to manufacturer’s instructions.

SH-SY5Y were differentiated with 10 μM retinoic acid (RA) (Sigma-Aldrich) for 3 days.

Primary hippocampal neurons were obtained from postnatal day (P) 0 B6/129 mice as previously described (Siano et al., 2019). At 48 h after plating cells have been transfected with Lipofectamine 2000 according to manufacturer’s instructions.

### Tau Seeding and Drug Treatment

Recombinant heparin-assembled P301S Tau fibrils were prepared as described by KrishnaKumar and Gupta (2017) with the following modifications: for protein purification we used the buffer A (50 mM MES pH 6,25, 0,5 mM DTT) and buffer B (50 mM MES pH 6,25, 0,5 mM DTT, 1 M NaCl) with the HiLoadTM 16/10 SP Sepharose^T*M*^ High Performance column (GE Healthcare Life Sciences); the protein was eluted using a linear gradient 0–100% of buffer B in six column volume; for protein aggregation 400 μg/ml heparin (Sigma Aldrich) has been added.

Cells were plated in glass bottom dishes as previously described and 1.2 μg of P301S Tau fibrils were delivered to cells with 2 μl of Lipofectamine 2000 transfection reagent diluted in 300 μl of Opti-MEM Reduced Serum Medium (Gibco). Cells were treated for 2 h, then DMEM low glucose, DMEM/F-12 or Neurobasal-A were added back to HeLa, SH-SY5Y or primary neurons, respectively. Scale up and scale down were performed as needed. The day after Tau seeding, cells have been treated with 1 μM PD-901 (sc-205427; Santa Cruz Biotechnology) or 6 μM D-JNKI-1 (HY-P0069/CS-5624; MedChemExpress) for 48 h in the presence of fibrils, while 10 μM STS (Cell signaling technology) or 200 nM OA (Cell signaling technology) were administered 72 h after Tau seeding for 1.5 h.

### Preparation of TritonX 100-Insoluble Fractions

Cells were lysed with lysis buffer (1% Triton-X 100 in PBS with protease and phosphatase inhibitors) and the extract was centrifuged at 16000 × *g* for 15 min. The supernatant was designated as Triton X-100 soluble fraction. The pellet was dissolved in the canonical lysis buffer (1% SDS; 1% Triton-X in PBS with protease and phosphatase), sonicated and boiled. This fraction was designated to be Triton X-100 insoluble fraction.

### Western Blot, Immunofluorescence and K114 Staining

For WB analyses, total cell extracts were prepared in lysis buffer supplemented with protease and phosphatase inhibitors. For each sample, 30 μg of each fraction has been loaded. Proteins were separated by 8% or 4–20% Tris-Glycine SDS-PAGE (Bio-Rad) and electro-blotted onto nitrocellulose membranes Hybond-C-Extra (Amersham Biosciences). Membranes were blocked (5% wt/vol non-fat dry milk) and incubated with the primary antibody (O/N, 4°C) and with HRP-conjugated secondary antibodies (1 h, RT). For IF experiments, cells were fixed with ice-cold 100% methanol for 5 min. Cell membranes were permeabilized (0.1% Triton-X100 in PBS) and samples were blocked (1% wt/vol BSA in PBS) and incubated with the primary antibody (O/N, 4°C) and with fluorophore-conjugated secondary antibodies (1h, RT). Slides were mounted with VECTASHIELD mounting medium (Vector Laboratories). Primary antibodies for WB were as follows: mouse anti-Tau (Tau5) 1:1000 (abcam); rabbit anti-pTau (Ser202, Thr205) 1:500 (Thermo Fisher Scientific); rabbit anti-pTau (Ser356) 1:500 (Thermo Fisher Scientific); mouse anti-pTau (Thr231) 1:500 (Thermo Fisher Scientific); mouse anti-GAPDH 1:15000 (Fitzgerald). Secondary antibodies for WB were as follows: HRP-conjugated anti-mouse or anti-rabbit Abs (Santa Cruz Biotechnology). Primary antibodies for IF: mouse anti-Tau (Tau-13) 1:500 (Santa Cruz). Secondary antibodies for IF were as follows: Alexa Fluor 633; Alexa Fluor 488 (Life Technologies). For K114 staining, cells were fixed and permeabilized as described above. Samples were incubated with 1 μM K114 (Sigma-Aldrich) for 10 min and slides were mounted with VECTASHIELD.

### Image Acquisition

Images were acquired with the TCS SL laser-scanning confocal microscope (Leica Microsystems) using a 63 × /1.4 NA HCX PL APO oil immersion objective. A heated and humidified chamber mounted on the stage of the microscope was used for live imaging experiments in order to maintain a controlled temperature (37°C) and CO2 (5%) environment during image acquisition. An Argon laser was used for ECFP (λ = 458 nm) and EYFP (λ = 514 nm), K114 (λ = 380 nm), a He-Ne laser for Alexa Fluor 633 (λ = 633 nm). White scale bar = 10 μm.

### FRET and Colocalization Analysis

For sensitized emission FRET experiments, the donor ECFP was excited at 458 nm and its fluorescence emission was collected between 470 nm and 500 nm (donor channel) and between 530 nm and 600 nm (FRET channel). The acceptor EYFP was excited at 514 nm and its fluorescence emission was collected between 530 and 600 nm (acceptor channel). The donor and acceptor fluorophores were excited sequentially in order to minimize the bleed-through between donor, acceptor and FRET channels. Bleed-through corrected FRET images were generated using Youvan’s method (Youvan et al., 1997): FRET index = I_*FRET*_ − BT_*D*_ × I_*D*_ − BT_*A*_ × I I_*A*_. I_*FRET*_, I_*D*_ and I_*A*_ are the images of the sample in the FRET, donor and acceptor channel after background subtraction, respectively. BTD and BTA are the contributions to FRET channels of donor and acceptor emission bleed-through, respectively. BTD parameter was determined by acquiring images in donor and FRET channels in cells expressing only the donor (transfected with the pECFP plasmid) and using the ImageJ plugin “FRET and Colocalization Analyzer” (Hachet-Haas et al., 2006). BTA parameter was determined by acquiring images in acceptor and FRET channels in cells expressing only the acceptor (transfected with the pEYFP plasmid) that were then subjected to same process of BTD in the ImageJ plugin. Typical values in our experimental conditions are BT_*D*_ = 0.1 and BT_*A*_ = 0.2. Normalized FRET (NFRET) images were obtained using the ImageJ software plugin “pixFRET” (Feige et al., 2005) by using: NFRET = F index/donor.

For colocalization experiments, images underwent background subtraction and were analyzed using Costes approximation method (Costes et al., 2004) in the ImageJ plugin “Coloc2.”

### Statistical Analysis

In Western blot, differences between means were assessed using non-parametric Mann-Whitney test or Kruskal-Wallis test followed by pairwise Mann-Whitney test. The corresponding quantification graphs have been obtained by normalizing each signal on the housekeeping gene and then by normalizing the signal of each phospho-epitope on total Tau. We calculated the fold changes between treated and untreated cells. Four experimental replicates have been performed. In FRET analysis, differences between means were assessed using Student’s t-test or one-way ANOVA followed by Tukey multiple comparisons test. In colocalization analysis, differences between means were assessed using Mann-Whitney test. All tests were performed using Origin (OriginLab, Northampton, MA).

In box-plots, values are expressed as the mean (square) ± SEM (box) and ± STD (whiskers); in bar-plots, values are expressed as the mean ± SEM. Significance is indicated as ^∗^ for *p* < 0.05, ^∗∗^ for *p* < 0.01, ^∗∗∗^ for *p* < 0.001 and ^****^ for *p* < 0.0001.

## Data Availability

All datasets generated for this study are included in the manuscript and/or Supplementary Files.

## Author Contributions

GS, CDP, VQ, and AC contributed conception and design of the study. GS, CDP, MCC, MV, IO, MC, and GM contributed the experimental investigation. GS, CDP, MV, and MCC contributed writing the original draft. GS, CDP, MCC, MV, and AC contributed writing, review, and editing. CDP and AC contributed funding acquisition.

## Conflict of Interest Statement

The authors declare that the research was conducted in the absence of any commercial or financial relationships that could be construed as a potential conflict of interest.

## Supplementary Material

FIGURE S1 CST aggregates are comparable to unlabeled Tau aggregates. **(A)** CST reporter cells have been visualized by live imaging in the acceptor channel (yellow) and by Immunofluorescence exploiting and the anti Tau antibody Tau13 (red). Magnification in the IF image highlights aggregates morphology. **(B)** Unlabeled Tau reporter cells have been visualized by Immunofluorescence exploiting the anti Tau antibody Tau13 (red). Magnification in the IF image highlights aggregates morphology.

FIGURE S2 The modulation of phosphorylation alters aggregation. **(A)** Colocalization of CST aggregates and K114 staining by confocal microscopy in cells treated with STS or OA. The CST signal is acquired in the YFP channel (yellow), K114 (magenta), white scale bar = 10 μm. **(B)** Fluorescence intensity of K114 positive spots. (^∗∗∗^*p* < 0.001 ANOVA one-way test).

FIGURE S3 The phosphorylation inhibition by PD-901 and D-JNKI-1 reduces aggregation. **(A)** Cellular viability in treated cells probed with different concentrations of drugs. **(B)** Cellular viability in Tau aggregated cells probed with drugs. **(C)** Colocalization of CST aggregates and K114 staining by confocal microscopy in cells treated with PD-910 or D-JNKI-1. The CST signal is acquired in YFP channel (yellow), K114 (magenta), white scale bar = 10 μm. **(D)** Fluorescence intensity of K114 positive spots. (^∗∗^*p* < 0.01, ^****^*p* < 0.0001 ANOVA one-way test).
