# Supplementary figures and images for: Identification of an ERK Inhibitor as a Therapeutic Drug Against Tau Aggregation in a New Cell-Based Assay
Source: Front Cell Neurosci. 2019 Aug 21;13:386. doi: 10.3389/fncel.2019.00386 (PMC6712076; doi:10.3389/fncel.2019.00386)

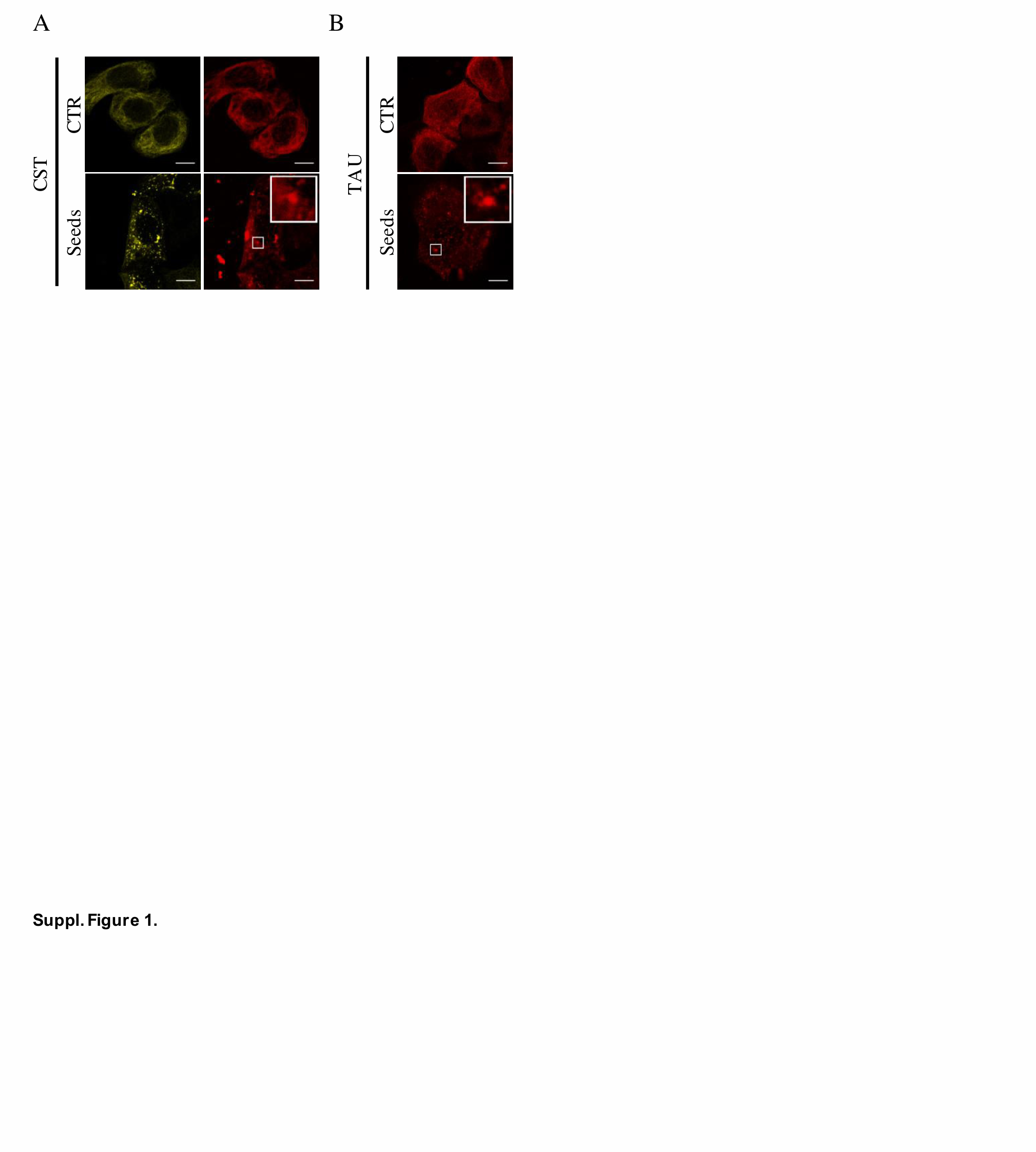

Supplement: FIGURE S1 — CST aggregates are comparable to unlabeled Tau aggregates. (A) CST reporter cells have been visualized by live imaging in the acceptor channel (yellow) and by Immunofluorescence exploiting and the anti Tau antibody Tau13 (red). Magnification in the IF image highlights aggregates morphology. (B) Unlabeled Tau reporter cells have been visualized by Immunofluorescence exploiting the anti Tau antibody Tau13 (red). Magnification in the IF image highlights aggregates morphology. [file Image_1.TIF]

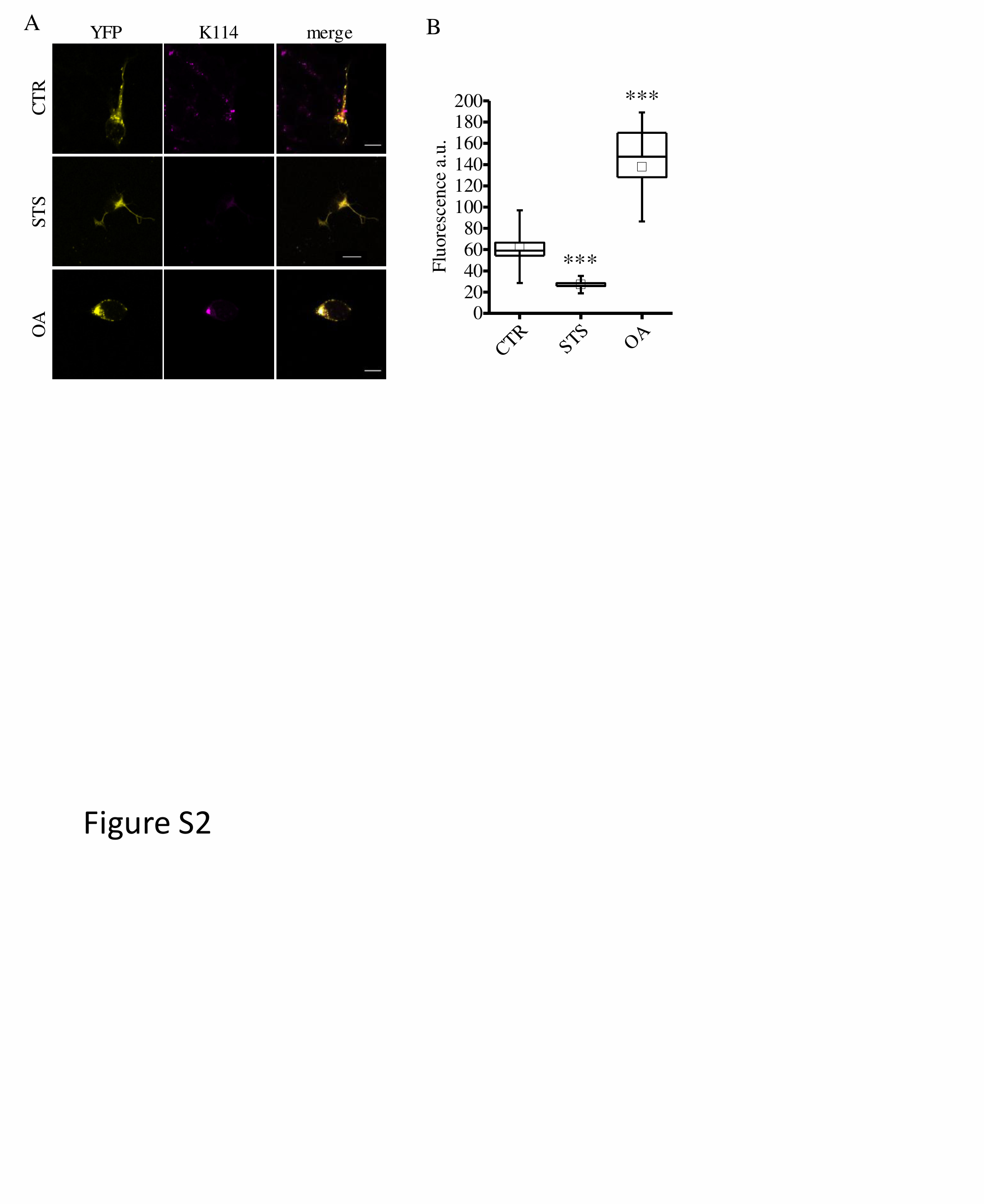

Supplement: FIGURE S2 — The modulation of phosphorylation alters aggregation. (A) Colocalization of CST aggregates and K114 staining by confocal microscopy in cells treated with STS or OA. The CST signal is acquired in the YFP channel (yellow), K114 (magenta), white scale bar = 10 μm. (B) Fluorescence intensity of K114 positive spots. (∗∗∗p < 0.001 ANOVA one-way test). [file Image_2.TIF]

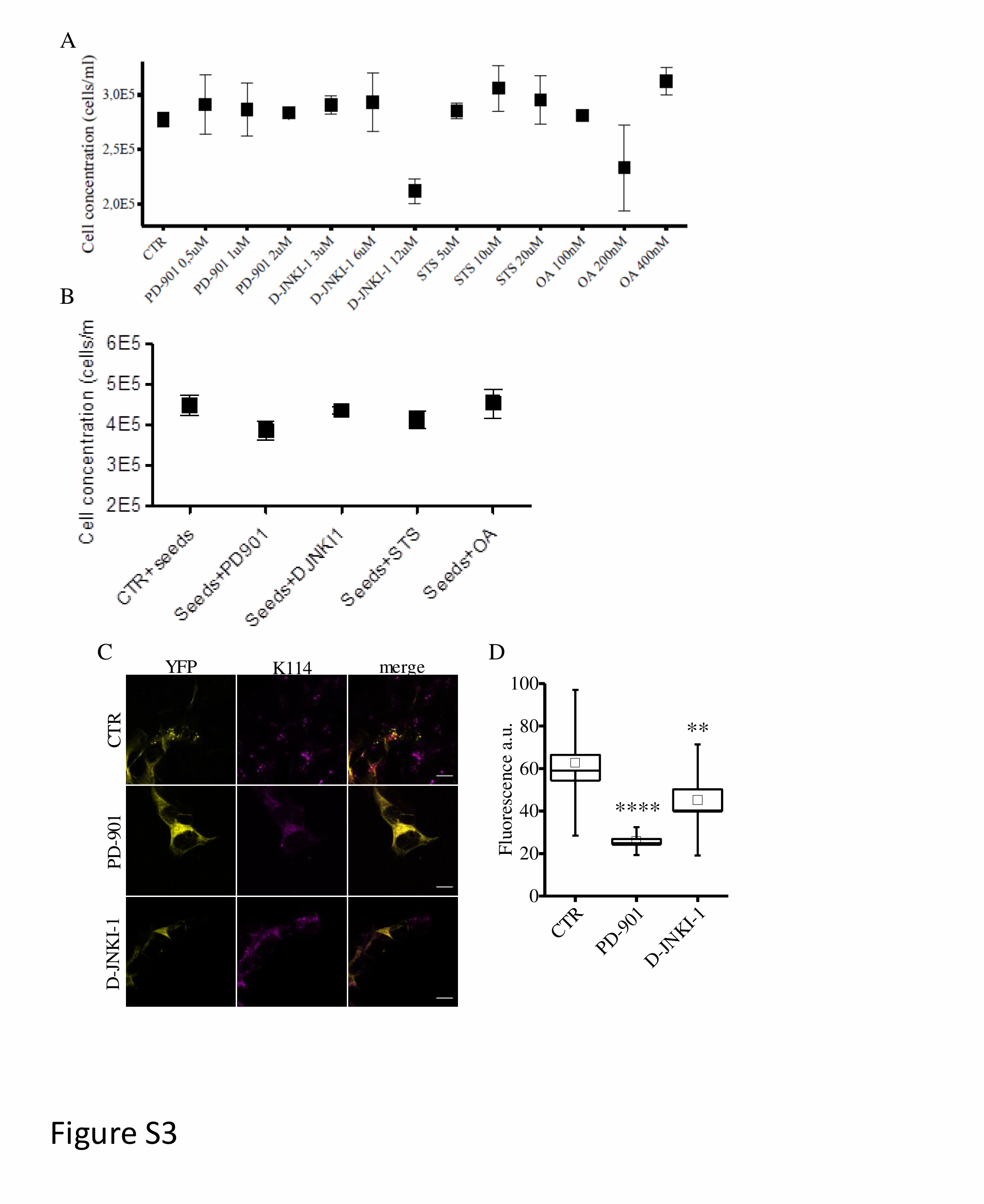

Supplement: FIGURE S3 — The phosphorylation inhibition by PD-901 and D-JNKI-1 reduces aggregation. (A) Cellular viability in treated cells probed with different concentrations of drugs. (B) Cellular viability in Tau aggregated cells probed with drugs. (C) Colocalization of CST aggregates and K114 staining by confocal microscopy in cells treated with PD-910 or D-JNKI-1. The CST signal is acquired in YFP channel (yellow), K114 (magenta), white scale bar = 10 μm. (D) Fluorescence intensity of K114 positive spots. (∗∗p < 0.01, ****p < 0.0001 ANOVA one-way test). [file Image_3.TIF]
